# Programmed Cell Death in Sepsis Associated Acute Kidney Injury

**DOI:** 10.3389/fmed.2022.883028

**Published:** 2022-05-17

**Authors:** Zhifen Wu, Junhui Deng, Hongwen Zhou, Wei Tan, Lirong Lin, Jurong Yang

**Affiliations:** ^1^Department of Nephrology, The Third Affiliated Hospital of Chongqing Medical University, Chongqing, China; ^2^Department of Nephrology, Chongqing Liangping District People's Hospital, Chongqing, China

**Keywords:** sepsis, acute kidney injury, apoptosis, pyroptosis, necroptosis, autophagy, sepsis-associated acute kidney injury

## Abstract

Sepsis-associated acute kidney injury (SA-AKI) is common in patients with severe sepsis, and has a high incidence rate and high mortality rate in ICU patients. Most patients progress to AKI before drug treatment is initiated. Early studies suggest that the main mechanism of SA-AKI is that sepsis leads to vasodilation, hypotension and shock, resulting in insufficient renal blood perfusion, finally leading to renal tubular cell ischemia and necrosis. Research results in recent years have shown that programmed cell death such as apoptosis, necroptosis, pyroptosis and autophagy play important roles. In the early stage of sepsis-related AKI, autophagy bodies form and inhibit various types of programmed cell death. With the progress of disease, programmed cell death begins. Apoptosis promoter represents caspase-8-induced apoptosis and apoptosis effector represents caspase-3-induced apoptosis, however, caspase-11 and caspase-1 regulate gasdermin D-mediated pyroptosis. Caspase-8 and receptor interacting kinase 1 bodies mediate necroptosis. This review focuses on the pathophysiological mechanisms of various programmed cell death in sepsis-related AKI.

## Introduction

Sepsis is a syndrome characterized by physiological, pathological and biochemical abnormalities caused by infection (1). It is characterized by severe systemic inflammation and multiple organ dysfunction (such as of the blood vessels, lungs, liver, kidney, heart and brain) due to the host's unregulated response to infection. Sepsis is an acute onset, severe condition with high mortality, and can involve multiple organs of the whole body. Multidrug resistant bacteria, drug side effects, and adverse events complicate the treatment of sepsis (2–5). Sepsis-associated acute kidney injury (SA-AKI) is the most common complication of sepsis, which significantly increases sepsis mortality (6). Sepsis complicated with AKI is multifactorial, and its specific mechanism has not been fully clarified. However, it has been proven that an excessive inflammatory response, intrarenal hemodynamic changes, coagulation dysfunction, microvascular endothelial dysfunction and renal tubular epithelial cell (RTECs) injury are all related to this process. A large number of studies have confirmed that the main injury mode of RTECs in acute renal injury caused by renal ischemia-reperfusion (I/R) is renal cell necrosis (7). For a long time, it was agreed that in SA-AKI, pathological changes in the kidney were characterized by ischemic necrosis of RTECs (8). With further study of sepsis-related acute renal injury, it was found that the reduction of renal blood flow was rarely confirmed in patients with sepsis (9–11), and there was no extensive tubular cell necrosis in autopsy analysis in the kidneys of patients with severe SA-AKI (12). In large animal models of septic shock, AKI can occur even if renal blood flow increases or remains unchanged (13, 14). In addition, pathological staining and protein level detection of renal tissue shows that the main pathological manifestation of SA-AKI is renal tubular cell apoptosis (11, 15–17). Necrosis, pyroptosis and autophagy-dependent cell death also play important roles. Necroptosis and ferroptosis are recently discovered modes of programmed cell death. At present, there are few reports on SA-AKI, and the roles of necroptosis and ferroptosis in SA-AKI need to be confirmed in further research.

**Figure 1:**
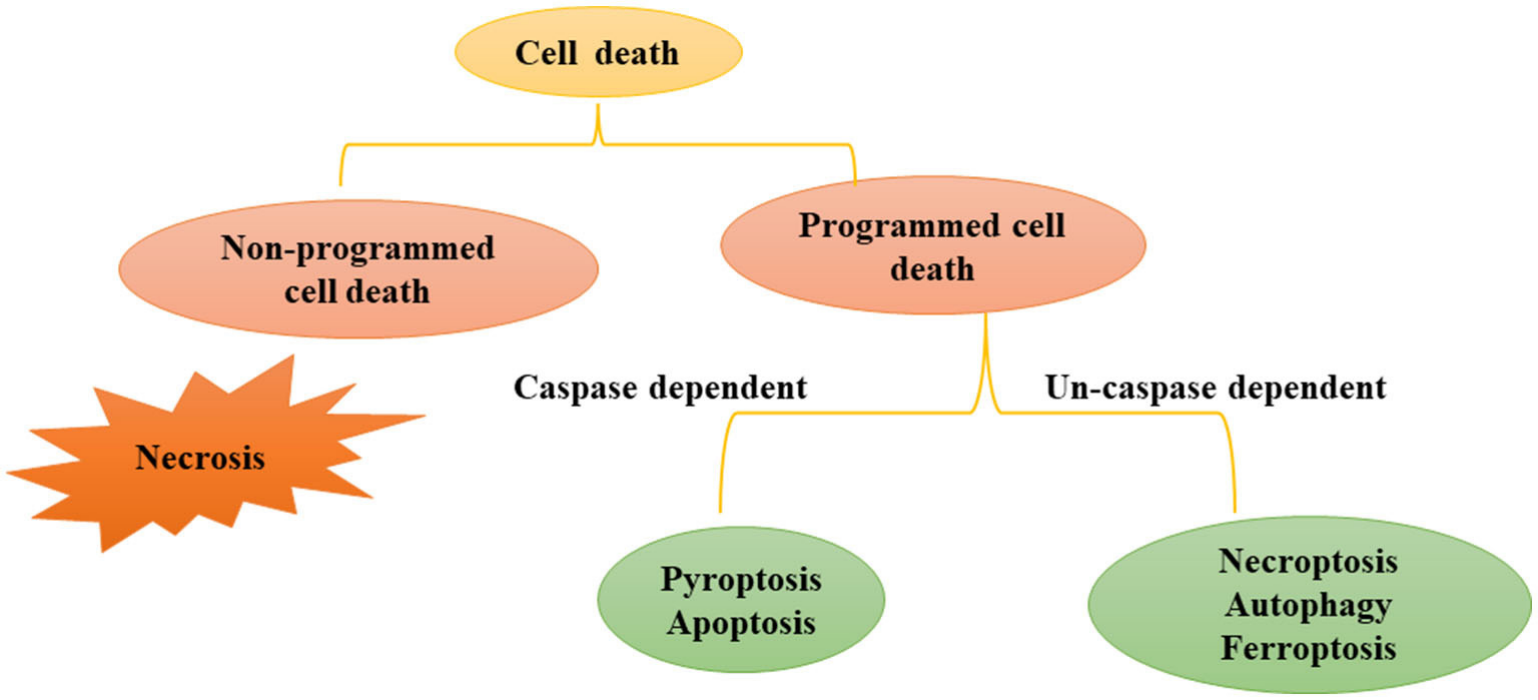
Modes of Cell death: programmed cell death and non-programmed cell death. Cell death includes programmed cell death and non-programmed cell death. Non programmed cell death represents cell necrosis. Programmed cell death can be divided into caspase dependent cell death (such as apoptosis and pyroptosis) and non-caspase dependent cell death (For instance necroptosis, ferrotosis, autophagy, etc.).

There are many types of cell death, that can be divided into programmed cell death (PCD) and non-programmed cell death (Figure 1). Non-programmed cell death is a biologically uncontrolled process, typically represented by cell necrosis. PCD involves tightly structured signal cascade reactions and molecularly defined effect mechanisms, including apoptosis, pyroptosis, necroptosis, ferroptosis, autophagy-dependent cell death, entotic cell death, netotic cell death, parthanatos, lysosome-dependent cell death, alkaliptosis and oxeiptosis (18). To date, studies have found that cell death modes such as necrosis, apoptosis, pyroptosis, necroptosis, ferroptosis, and autophagy-dependent cell death play an important role in the pathophysiological process of SA-AKI. In this review, we will introduce the definition, epidemiology and pathophysiological mechanisms of various types of programmed cell death in SA-AKI.

## Epidemiology

Sepsis is a global public health challenge (19). According to statistics, the survival rate of sepsis is only 30%. According to statistics of the World Health Organization, at least 6 million patients die of sepsis infection every year. Among the complications of sepsis, AKI is the most common and serious, and its mortality is the highest (6, 20, 21). Renal failure occurs in 30–50% of patients with sepsis, and the mortality of patients with AKI is 45–70%. Those who survive have a significantly increased risk of developing chronic kidney disease (20, 22–24). According to statistics, more than 60% of sepsis cases are related to AKI, and about 50% of AKI cases are related to sepsis (25, 26). Despite timely and active intervention, the mortality rate is still 45–70% (27). However, the exact cause of sepsis leading to this injury is unclear. The pathogenesis of SA-AKI is extremely complex, including inflammation, hemodynamics, microvascular dysfunction and renal tubular injury. Although some progress has been made in this field, there is still no standardized and satisfactory treatment strategy for SA-AKI, and specific and effective treatment methods are lacking, apart from blood purification and supportive treatment (28–30). Therefore, it is urgent to further explore the exact mechanism of SA-AKI, which can provide new insights for clinical treatment (31).

## Programmed Cell Death Modes in SA-AKI

At present, the forms of cell death related to AKI can be roughly divided into apoptosis, pyroptosis, ferroptosis, necroptosis, and autophagy-dependent cell death, necrosis (32–36). The occurrence of SA-AKI involves a variety of cell death pathways. Although the molecular mechanisms of these pathways differ, they are interrelated. Therefore, in the following chapters, we will briefly describe various cell death pathways and their relationships in SA-AKI.

## Apoptosis in SA-AKI

**Figure 2:**
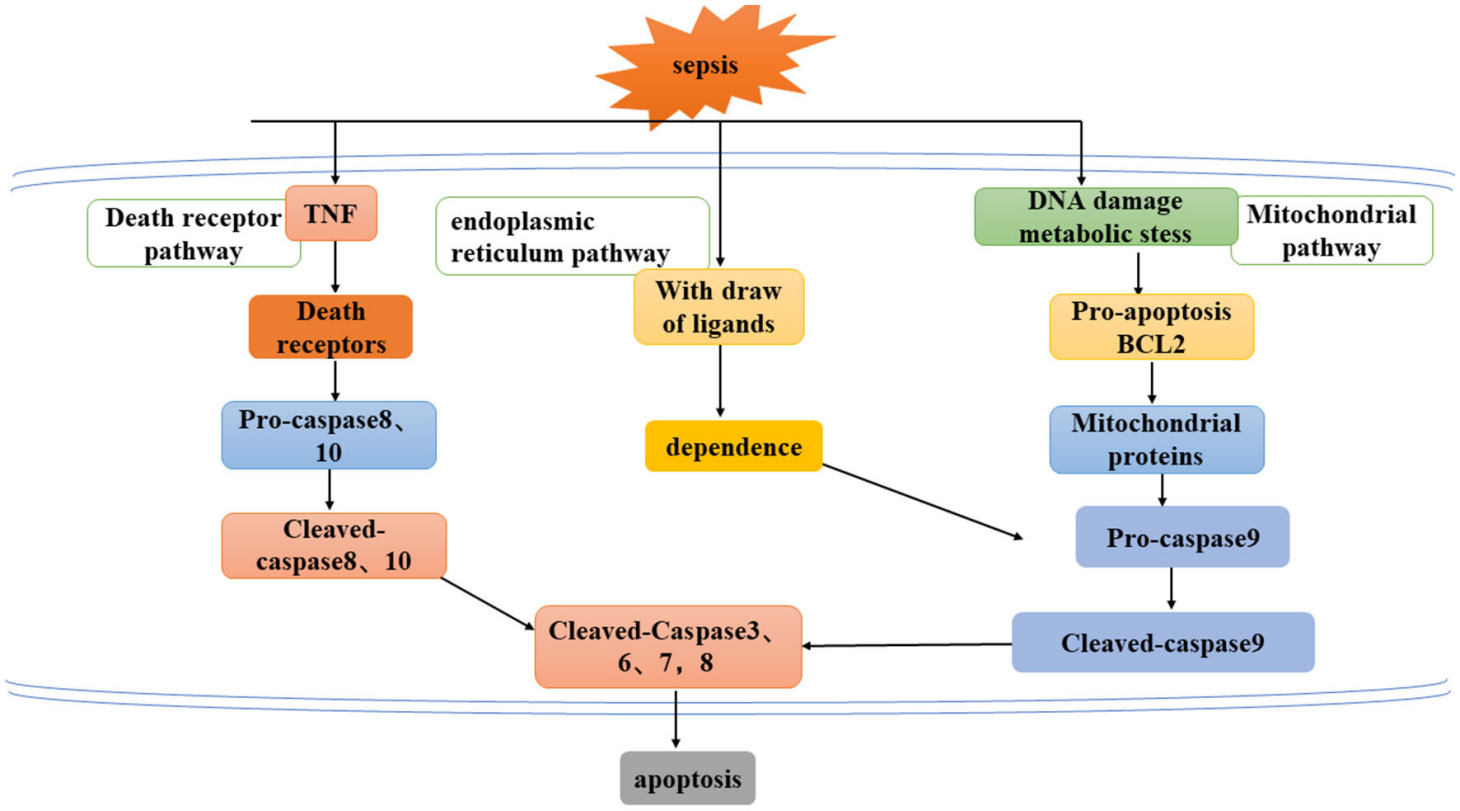
Three ways to mediate apoptosis: exogenous pathway mediated by death receptor, endogenous pathway mediated by mitochondria, and endoplasmic reticulum pathway.

Apoptosis refers to a series of programmed cell death processes that activate caspase cascade apoptosis under the regulation of apoptotic genes. This process is often accompanied by chromatin condensation, DNA breakage, phosphatidylserine valgus, and the emergence of apoptotic bodies (18, 37). At present, there are three known pathways of apoptosis (Figure 2) an exogenous pathway mediated by death receptors, an endogenous pathway mediated by mitochondria, and an endoplasmic reticulum pathway.

Sepsis-related acute renal injury was initially recognized as renal tubular cell ischemia and necrosis caused by insufficient renal blood perfusion owing to sepsis. Therefore, for a long time, treatment was focused on increasing perfusion pressure and renal blood flow. However, a series of animal experiments and human data showed that most patients with sepsis-related acute renal injury do not have decreased renal blood flow perfusion. Interestingly, normal or increased renal blood flow was found in animal models of sepsis and in patients with sepsis (11, 15, 37–39). Lerolle et al. found that apoptotic cells were observed in the proximal and distal tubules of all patients with septic shock and anuria AKI through renal biopsy after death, which was compared with patients who died of non-infectious shock without renal injury in the ICU and patients with trauma without renal injury, Renal tubular cell apoptosis and capillary leukocyte infiltration were increased significantly, which strongly proved that apoptosis plays an important role in the pathogenesis of septic shock AKI (11). Similar results were obtained by Aslan et al. (17). However, in animal models of sepsis, pathological manifestations of the kidney are different from those of human patients, as shown in Table 1. The reasons for this may be: (1) in the animal models, especially mouse model, the body is small and the whole body blood circulation is rapid, resulting in inconsistent pathological damage between human patients and animals; (2) human specimens are collected after the patients' death, and blood circulation stops after the patient's death, leading to rapid organ death (42, 43).

**Table 1:** Modes of sepsis-associated acute kidney injury(SA-AKI).

**Type**	**Pathophysiology**	**Hemodynamics**	**References**
Patients with SA-AKI	(1) Pathological damage of renal tubules is not obvious, focal, and the glomerulus was basically intact; (2) massive inflammatory cell infiltration (glomerulus and tubulointerstitium); (3) apoptosis (3% tubular cells, almost no glomerulus).	Normal or increased renal blood flow	(12, 17)
Rodent model of sepsis related renal injury (Rat, Mouse)	(1) Renal cortex and interstitial edema with a large amount of inflammatory cell infiltration. (2) Renal tubular epithelial cells are swollen, vacuolar degeneration, necrosis and abscission. (3) Dilatation of renal tubular sac and formation of tubular shape. (4) Glomerular capillaries and medullary capillaries are not clear.	Normal or increased renal blood flow	(40, 41)
Mammal (pig, sheep)	Renal tubular cells show vacuolization and injury to cellular brush borders but no evidence of necroptosis.	Normal or increased renal blood flow	(41)

Mariano et al. treated renal tubular cells and podocytes with plasma from patients with severe burn and SA-AKI, and apoptosis was significantly increased compared with patients without AKI. This indicates that circulatory system factors lead to renal apoptosis (16, 40). Thus, the pathogenesis of sepsis-related AKI is complex, involving multiple factors, and apoptosis is dominant (11, 15–17). However, the specific mechanism of initiating apoptosis is not clear.

Lipopolysaccharide (LPS), a well-known circulating endotoxin and a component of the bacterial cell wall, is the main cause of septic shock and may lead to acute renal failure by directly inducing apoptosis of renal tubular cells through systemic cytokine release (40, 41). Guo et al. emphasized the role of LPS in AKI-induced apoptosis of renal tubular cells in mouse experiments (44). Intervention with caspase-3 inhibitor, could protect renal tubular cells from acute renal failure caused by LPS. Interestingly, in that model, caspase-3 inhibitors could not only prevent apoptosis, but could also reduce renal inflammation, confirming that caspase plays an important role in renal tubular cell apoptosis (45). In addition to epithelial cell injury, macrophages and endothelial cells are also a target in sepsis. Endothelial cell injury destroys microvascular blood flow, resulting in renal hypoperfusion, hypoxia and epithelial cell ischemia. In sepsis, macrophages can cause spontaneous death while scavenging microorganisms, but excessive apoptosis of macrophages may inhibit immune function and aggravate renal damage (46). The activation of glomerular endothelial cells increases vascular permeability. In gram-negative sepsis, LPS and tumor necrosis factor (TNF-α) both induced apoptosis of glomerular endothelial cells, in renal endothelial cells, TNF-α Receptor 1 induces apoptosis *via* a caspase-8-dependent pathway (47), and may promote sepsis-induced AKI *in vivo* (44, 48). In addition, Bannerman et al. proposed that LPS can also directly aggregate Fas-related death domain (FADD) and TNF receptor related death domain (TRADD) through the death effector domain (DED), thereby inducing the cleavage of Caspase-3, 6,7, and 8, apoptosis occurs finally (49). The activation of Caspase-3 signaling leads to the inhibition of protein hydrolysis and irreversible cell death. The translocation of cleaved caspase-3 into the nucleus leads to substrate cleavage, DNA degradation, and protein modification, and finally leads to the emergence of apoptotic bodies. Endotoxin stimulation and/or oxygen restriction leads to the production of reactive oxygen species (ROS), which can initiate apoptosis. Excessive ROS can stimulate p53 and induce apoptosis. B-cell lymphoma-2-associated X protein (Bax) is activated by ROS and/or p53 and is transferred to the outer membrane of mitochondria, resulting in the leakage of cytochrome c (Cytc), which is a strong inducer of apoptosis (50).

It can be seen that apoptosis plays a vital role in SA-AKI. Blocking apoptosis is expected to reduce mortality patients with SA-AKI and block the progression to chronic kidney disease (CKD). Recent study reported that apoptosis belongs to immune silent death, and no damage related molecular patterns (DAMPs) was released by apoptotic cells (51, 52). Studies have confirmed that necroptosis and ferroptosis play a very important role in the pathophysiological progress of SA-AKI (53).

## SA-AKI and Necroptosis

**Figure 3:**
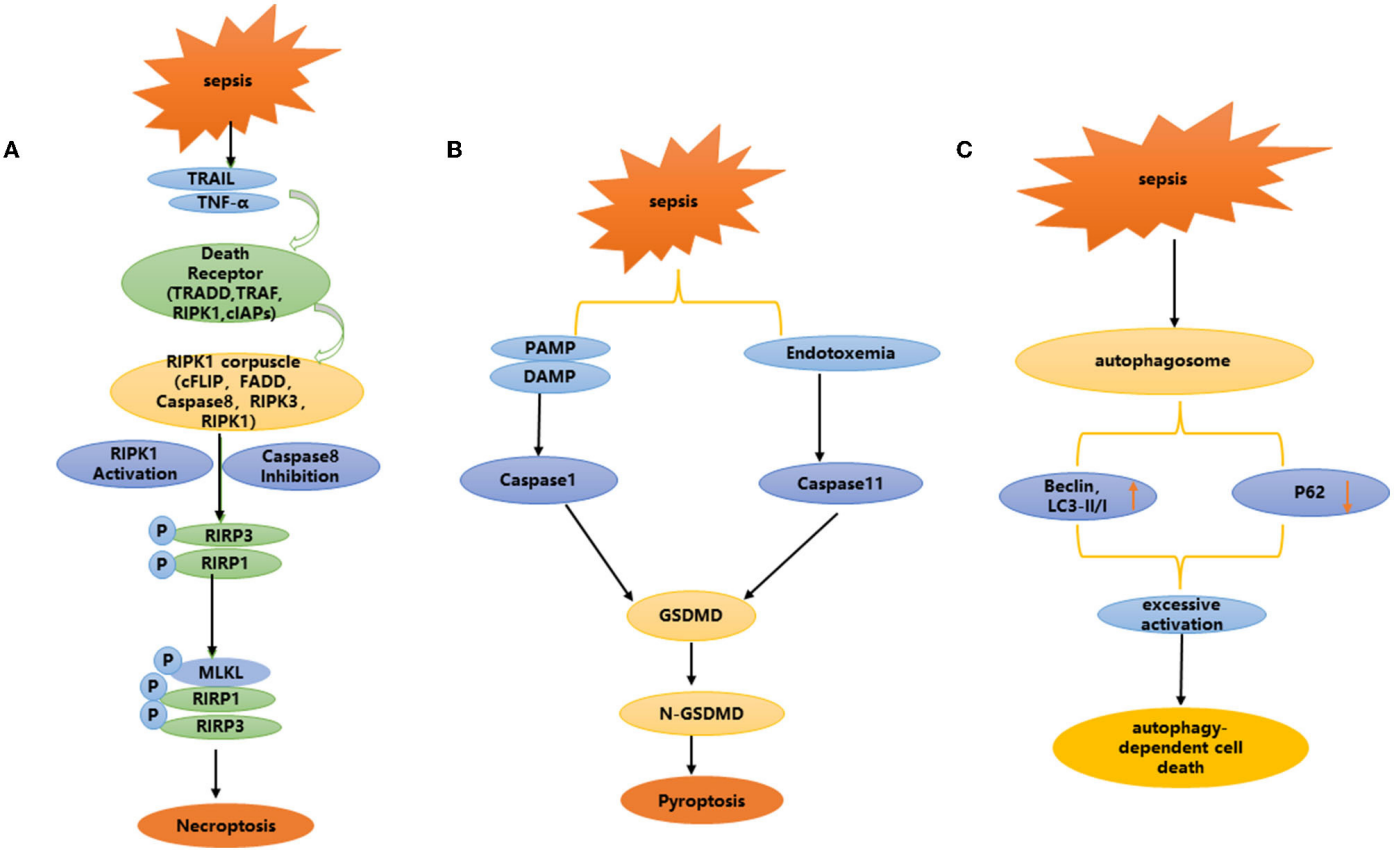
**(A–C)** Non-apoptotic programmed cell death: necroptosis, pyroptosis, and autophagy dependent cell death.

Necroptosis is a recently discovered programmed cell death mode that is morphologically different from apoptosis. Studies have confirmed that necroptosis can cause the death of renal cells, which is closely related to inflammation (53). It is characterized by plasma membrane rupture, cell content leakage and organelle swelling (54–56). Necroptosis is not dependent on caspase, but is involved in regulation through multiple cellular signal transduction proteins, including receptor interacting protein kinase 1 (RIPK1), receptor interacting protein kinase 3 (RIPK3) and its downstream target mixed lineage kinase domain like (MLKL) (57) (Figure 3A). Studies have shown that many cell mediators can activate the necroptosis pathway, such as death receptors, interferons, Toll like receptor (TLRs), and RNA or DNA sensors (58).

The activation of RIPK1 is widely considered to be the main mediator of necrotic apoptosis mainly through its binding to TNF receptor (TNFR) (59). RIPK1 further activates RIPK3 through the homologous interaction motif (RHIM) and then activates MLKL (60–62). After activation, oligomers of phosphorylated mixed lineage kinase-like (MLKL) protein are formed and transferred to the plasma membrane. This structure leads to cell swelling, rupture and the release of damage-associated molecular patterns (DAMPs) (62, 63). The pathophysiology of septic AKI is different from that of other AKI models. After systemic injection of lethal doses of lipopolysaccharide (LPS), no significant RTECs necrosis is observed in septic kidneys (64). Therefore, the role of necroptosis in septic AKI has attracted interest in current research. *In vitro* experiment, Sureshbabu et al. (65) showed that the expression levels of phosphorylated RIPK3 (p-RIPK3), RIPK3 and p-MLKL increased in a time-dependent manner under LPS stimulation in human renal proximal tubular cells (HK-2 cells). In mice undergoing CLP surgery, the expression levels of p-RIPK3, RIPK3, and MLKL in renal tissue were higher 6 h after treatment. These results suggest that RTECs necroptosis is also involved in the occurrence and development of SA-AKI. However, there are few studies on the necroptosis of endothelial cells and RTECs during sepsis. Here, we propose to emphasizing the necroptosis regulation of renal vascular endothelial cells and RTECs.

Endothelial cells and macrophages also play an important role in SA-AKI necrotic apoptosis. In addition, programmed cell necrosis was also observed in cisplatin, I/R and contrast medium induced nephrotoxicity. At present, there are few reports on necroptosis in sepsis, especially sepsis related AKI. Further research is needed to deepen our understanding and provide a new direction for clinical treatment. In addition, necroptosis is involved in various other pathological conditions, such as I/R injury, nonalcoholic steatohepatitis and atherosclerosis (66).

## SA-AKI and Pyroptosis

Pyroptosis is a type of pro-inflammatory programmed cell death. It is considerably different from apoptosis and autophagy in cell morphology and function. It is activated by members of the inflammation related caspase family, which cleave gasdermin family proteins to expose their N-terminus and transfer to the cell membrane, resulting in cell membrane damage and 1.1–2.4 nm perforation. By changing the osmotic pressure inside and outside the cell, the cell becomes swollen and ruptured, and membrane integrity is lost, which eventually leads to the release of inflammatory factors [including interleukin-1 (IL-1) and IL-18] mediated by cell collapse and pyroptosis (67, 68). However, during this process, the structure and function of mitochondria remain intact (69).

Pyroptosis is involved in the occurrence and development of a variety of diseases, especially related to the occurrence and development of kidney diseases. Focal death is mainly involved in renal disease in two ways: *via* the caspase-1-mediated classical focal death pathway and the caspase-11 mediated non-classical focal death pathway (Figure 3B). Caspase-1 mediated classical focal death is a regulated form of cell death, which depends on the activation of caspase 1.When the host is infected and generates a danger signal, such as damage-associated molecular patterns (DAMPs), pathogen-associated molecular patterns (PAMPs), nod like receptors (NLRs) recognize these signals and trigger focal death (70, 71). NLRs, usually involve NLRP3 or NLRP4, and NLRP3 is one of the most important intracellular receptors, that can be expressed by NF-κ B transcription induction. Activation of the NLRP3 inflammasome plays a key role in focal death (72–75). After recognizing the ligand, the adaptor protein apoptosis-associated speck-like protein (ASC) is captured by NLRP3, while activating caspase-1, resulting in inflammatory factors such as IL-18 and IL-1β (76–78). Cleaved-caspase-1 cleaves the N-terminal sequence of gasdermin D(GSDMD), binds to the membrane, and forms membrane pores, resulting in cell swelling, membrane rupture and cell death. In addition, the production of reactive oxygen species (ROS), is a potential trigger for NLRP3 inflammasome assembly (79). Due to infection, inflammation or mitochondrial dysfunction, the level of mitochondrial reactive oxygen species (mtROS) increases, and mitochondrial DNA is released into the cytoplasm in the form of oxidation. This occurs through direct interaction between oxidized mitochondrial DNA (ox-tmDNA) and NLRP3, triggering assembly and activation of the NLRP3 inflammasome (80–82). In a rat model of sepsis, Yang et al. found that the caspase-1 inhibitor AC-YVAD-CMK significantly reduced the expression of GSDMD in renal tissue, and protected against acute renal injury caused by sepsis by blocking the expression of NLRP1 inflammatory body, reducing the scorch death of RTECs induced by the inflammatory body, increasing antioxidant enzyme activity, and weakening oxidation products (83).

In the non-classical pyroptosis pathway, caspase-4, 5, and 11 can be directly activated after interacting with bacterial LPS, resulting in the cleavage of GSDMD, independent of the activation of NLRP3 and caspase-1 (84–86). Caspase-11 is an intracellular cysteine protease that mediates the response of host cells to gram-negative bacterial pathogens and sepsis. Host cells sense LPS in gram-negative bacteria through a non-standard inflammasome pathway, which eventually leads to caspase-11 activation and pyroptosis (76, 78, 87–89). A series of studies have shown that caspase-11 plays a key role in pyroptosis (76, 78, 87–92). Ye et al. used LPS induced sepsis to produce soluble death of RTECs, accompanied by increased expression of focal death-related proteins caspase-11 and GSDMD. Through caspase-11 gene knockout, those authors further confirmed that caspase-11 mediated focal death plays an important role in SA-AKI. LPS induced focal death-related protein expression increases and weakens the focal death of RTECs induced by caspase-11 (93).

In SA-AKI, pyroptosis is also observed in macrophages and endothelial cells in addition to renal tubular cells (94, 95). Compared with *in vitro* experiments, the *in vivo* experiments show more severe focal death in renal tubular cells. Current evidence suggests that inhibition of pyroptosis signaling can improve SA-AKI in mice.

## SA-AKI and Ferroptosis

Ferroptosis is a unique programmed cell death mode discovered by Stockwell et al. (96) (Figure 3C). It is characterized by excessive accumulation of iron, enhanced lipid peroxidation and ineffective clearance of lipid peroxide (97), mainly characterized by smaller mitochondria and increased membrane density (98), without nuclear condensation, DNA fragmentation and caspase activation. Glutathione peroxidase 4 (GPX4) and prostaglandin endoperoxide synthase 2 (PTGS2) are well-known markers of ferroptosis. SystemXc- (SXC), glutathione (GSH) metabolism regulating GPX4 activity and ROS production, and participate in the regulation of ferroptosis (96–98).

Recent studies have shown that ferroptosis is involved in the occurrence and development of a variety of diseases, including degenerative brain disease, and injury to the heart, liver, intestines, and other organs (99). Xiao et al. reported that after cecal ligation and puncture (CLP), the expression of GPX4 decreased significantly and the expression of PTGS2 increased. This was the first confirmation that ferroptosis was involved in SA-AKI (100), but the mechanism of ferroptosis in SA-AKI needs to be further studied.

Although there are few reports on ferroptosis in SA-AKI, it is worth noting that ferroptosis was first found in the kidney, and ferroptosis is closely related to inflammation. It is necessary to further study ferroptosis in SA-AKI.

## SA-AKI and Autophagy-Dependent Cell Death

Autophagy, as one of the main models of cell death, has been considered key to maintaining intracellular homeostasis and the stress response. Autophagy plays an important role in maintaining and promoting cell survival and metabolism (101–103). Autophagy is also a degradation system *via* which intracellular pathogens, damaged or long-lived proteins, and dysfunctional organelles are encapsulated within autophagosomes and are degraded in lysosomes (104).

Autophagy plays an important role in maintaining intracellular homeostasis in SA-AKI (105). LPS, a mediator of gram-negative bacterial sepsis, induces autophagy in RTECs *in vivo* and *in vitro* (106, 107). Wu et al. found that the expression levels of autophagy related proteins Beclin1 and LC3 II/I increased significantly and the expression level of autophagy substrate p62 decreased in a mouse model of polymicrobial sepsis induced by CLP (108). Compared with mice in CLP group, there was less renal injury in CLP + 3-MA (autophagy inhibitor) group, and the postoperative survival rate was significantly higher than that in the CLP group, The autophagy inhibitor chloroquine increases the sensitivity of mice to the lethal effect of CLP, indicating that sepsis can activate autophagy *in vivo* (108, 109). After determining endotoxemia, the administration of ticrolimus inhibitor can prevent the occurrence of LPS induced AKI by inducing autophagy. These results suggest that autophagy is activated and provides protection in sepsis associated acute renal injury (106, 107). Other studies have found that in mice receiving CLP, renal autophagy was enhanced in the early stage (6 h), indicating that the accumulation of LC3-II and autophagic flow increased, and then decreased at a later time point (24 h). Further induction of autophagy by rapamycin can improve renal function and apoptosis of RTECs in CLP mice (110). In a study using a rat model of CLP induced sepsis, autophagy initially increased at 4 h and then began to decrease after 9 h. The decrease of autophagy led to the apoptosis of proximal renal tubular cells during sepsis (111). This shows that autophagy can protect the body from sepsis induced AKI (108).

Leventhal et al. (106) and Mei et al. (107) used renal proximal tubular epithelial cell specific Atg7 gene knockout mice and found that PTAtg7 knockout mice had more severe renal dysfunction and parenchymal injury than wild-type mice, and there was evidence of increased IL-6 and STAT activation in renal tissue caused by LPS (106). *In vitro* experiments showed that autophagy function was impaired and IL-6 production was enhanced in Atg7 knockout RTECs, which was related to LPS (106). These studies showed that Atg7 gene knockout enhanced TNF- α induced cell death, whereas the autophagy activator rapamycin inhibited the death of renal tubular cells *in vitro* (111). These studies suggest that enhanced autophagy provides a general renal protective mechanism in septic AKI by reducing apoptosis, inflammation and oxidative stress (54).

Autophagy dependent cell death is a regulated form of cell death, which depends on the autophagy mechanism, and may occur in a specific environment, contributing to the pathogenesis of disease (68, 112).

In addition to participating in regulated cell death, autophagy can affect other basic processes associated with aging or disease, including the regulation of inflammation, innate immunity and host defense (113, 114). Studies have shown that the autophagy mechanism critical for the degradation of cell death components and regulatory factors. Autophagy also plays a role as a scaffold of necrosis. Inhibiting lysosomal fusion can aggravate cell death. Importantly, these two functions can lead to competitive effects, that is, inhibiting or increasing the possibility of cell death.

## SA-AKI and Necrosis

In the early study of SA-AKI, it is agreed that SA-AKI is the change of renal hemodynamics caused by septic blood, which leads to renal ischemia and hypoxia and acute tubular necrosis (ATN) (64). The Pathological results showed that ATN was discontinuous distributed in some renal biopsy tissues of some septic patients, and the morphology of most patients was stage 2 ATN, vacuolization, renal tubular edema, epithelial flattening and some apoptotic renal tubular cells (17). However, a series of subsequent studies confirmed that Multiple programmed cell death play a more important role in SA-AKI. This paper mainly expounds the role of programmed cell death in SA-AKI, and does not make too much statement for necrosis.

## Relationship Between Various Types Programmed Cell Death

Apoptosis, pyroptosis, necroptosis, and autophagy are involved in the pathophysiological progress of SA-AKI, each with unique morphological, cell biological and biochemical characteristics. These modes exist simultaneously in SA-AKI (Table 2). The latest research reports that ferroptosis also plays an important role in SA-AKI. For example, apoptosis and necrosis are found in renal tissue in SA-AKI (11). Guo et al. (115) and Liu et al. (116) found that apoptosis and pyroptosis coexist in CLP mice. Li and Liu confirmed the same results (117, 118). Yu et al. (108) found that autophagy existed in a CLP mouse model, in the early stage, autophagy was stronger, and gradually weakened over time. In addition, Chen et al. reported that RIPK3 mediated necroptosis and GSDMD mediated pyroptosis play a synergistic role in sepsis, aggravating septic inflammatory response and tissue damage (119). These evidences suggests that all types of programmed cell death may occur in the kidney at the same time.

**Table 2:** Modes cell death.

**Type**	**Morphological features**	**Biochemical features**	**Cell morphology**	**Cell membrane integrity**	**References**
Apoptosis	Chromatin condensation, DNA breakage, phosphatidylserine valgus, emergence of apoptotic bodies,	Pro-apoptotic BCL-2 family members, caspase-activation, cleavage of various caspase substrates	The cell membrane shrinks and the cell volume becomes smaller	The cell membrane remained intact until apoptotic bodies were formed	(19, 39)
Pyroptosis	Inflammation related caspase family members activate, cleave gasdermin family proteins to expose their N-terminal and transfer to the cell membrane, resulting in cell membrane damage and 1.1–2.4 nm perforation; Changes in intracellular and extracellular osmotic pressure, cell swelling and rupture, loss of membrane integrity and release of inflammatory factors.	Cativation of caspase-1,-3,-11 and GSDMD, GSDMD cleavage GSDMD-N induced pore formation release.	The cell volume becomes larger and deformed	Destruction of cell membrane structure	[71, 171]
Autophagy	Cell autophagy is a process of swallowing its own cytoplasmic proteins or organelles and encaspsulating them into vesicles, fusing with lysosomes to form autophagic lysosomes and degrading the contents, so as to meet the metabolic needs of cells and the renewal of some organelles.	Accumulation of autophagic vacuoles, vacuolization of the cytoplasm, no chromatin condensation. Autophagy-related (.atg) family of gene encoded proteins, LC3-I to LC3-II conversion and cleavage of p62.	Produce cavitation and form	The cell membrane structure is intact	[105, 172, 173]
Necroptosis	Necroptosis is programmed cell death independent of caspase, which is regulated by multiple cell signal transduction proteins, including RIPK1, RIPK3 and its downstream target MLKL. It is characterized by the rupture of plasma membrane, leakage of cell contents and swelling of organelles.	Cytoplasmic swelling, loss of plasma membrane integrity, cytoplasmic organelle swelling. RIPK1, RIPK3, MKL, phosphorylation and ubiquitination of RIPK1, formation of necrotic complex in cytoplasm, phosphorylation and activation of MLKL, effectors of caspase, production of reactive oxygen species and release of DAMPs.	The cell volume becomes larger and deformed	Destruction of cell membrane structure	(54–58)
Ferroptosis	Ferroptosis is a newly discovered type of programmed cell death. It is characterized by excessive iron accumulation, enhanced lipid peroxidation and ineffective clearance of lipid peroxide. It is mainly manifested in smaller mitochondria and increased membrane density, without nuclear condensation, DNA breakage and caspase activation.	Glutathione depletion, glutathione peoxidase 4activity decreased; lipid oxides cannot be reduced, and divalent iron ions oxidize lipids to produce a large amount of reactive oxygen species, resulting in iron-dependent cell death.	The cell volume becomes larger and deformed	Destruction of cell membrane structure	[98, 99, 174]

Apoptosis, autophagy, pyroptosis, and necroptosis also interact. With many interrelated events among them. The most typical is caspase-8, which is involved in different cell death pathways. Caspase-8 is thought to interact with ASC to activate apoptosis (108, 120) and strengthen the apoptotic cascade leading to the inhibition of necroptosis (121). Fritsch et al. (122) found that the expression of inactive caspase-8 led to the death of mouse embryos, which may be due to the enhancement of necroptosis and focal death. MLKL deficiency could rescue the cardiovascular phenotype, but unexpectedly led to perinatal death in caspase8 +/+ mice, suggesting that focal death occurs when apoptosis and necrotic apoptosis are inhibited simultaneously (122). In addition, in caspase-8+/+ MLKL–/– ASC–/– or caspase-8+/+MLKL–/– caspase-1–/–mice, early death and embryo lethality were completely prevented. This suggests that caspase-8 represents a molecular switch that controls apoptosis, necroptosis and focal death (122).

Apoptosis is a type of cell death, that occurs by activating caspases. Autophagy and apoptosis are mutually regulated. Hou et al. (123) found that autophagy is involved in the regulation of cysteine enzyme activity. In Bax–/– HCT116 cells, treated together with cell death stimulator (for example: TNF-related apoptosis-inducing ligand: TRAIL), knockdown Beclin-1 or vps34 at the same time, and inhibits the occurrence of autophagy. Study results showed that the expression of cleaved caspase-8 increased significantly in the cytoplasm. A small amount of activated caspase-8 can be eliminated by lysosomal degradation and can inhibit the occurrence of apoptosis. Further studies showed that the transition from autophagy to apoptosis mainly depends on caspase-8, because knockdown of other caspases in these cells did not prevent autophagy from inhibiting the observed apoptotic transition. In addition, Fortunato et al. found that inhibition of autophagy can induce caspase-8 activity and promote apoptosis, and impaired fusion of autophagosomes and lysosomes can also lead to apoptosis (124). CD95/Fas mediated apoptosis is enhanced by autophagic degradation of protein tyrosine phosphatase non-receptor type 13 (PTPN13). In the same cells, autophagy inhibits apoptosis through TRAIL death receptors (125).

The relationship between autophagy and necroptosis is complex. Various studies have shown that autophagy flux or lysosomal dysfunction is key to the spread of necroptosis. Necroptosis usually occurs simultaneously with autophagy activation (54, 126). Knockout of Atg5, Atg7, or Beclin 1 can prevent necroptosis (127, 128), whereas depletion of Atg16L1 or Atg7, which reduced autophagy flow, can promote necroptosis (129, 130). Recently, autophagy has been shown to be involved in the conversion of complexes containing TRIF, RIPK1, RIPK3, and Z-DNA-binding protein 1 (ZBP1) (131). Deletion of autophagy receptor optineurin (OPTN) can lead to RIPK3-dependent necroptosis (112, 132). Rapamycin treatment and increased autophagy flux reduce RIPK1 expression and protect photoreceptor cells from Zvad-induced necroptosis (133). The negative regulator of necroptosis is caspase-8 (134). Active caspase-8 can cut RIPK1 and RIPK3, and then block necroptosis (135–137).

Ferroptosis is also regulated by autophagy. The increase in ferritin phages allows for the accumulation of free iron and promotes iron sagging (138). Knocking down regulatory factors of autophagy, such as Atg3, Atg5, Atg7, or Atg13, can inhibit ferroptosis (138, 139). A study by Zhang et al. showed that the kinase inhibitor sorafenib promoted the increase of autophagy flux and ferritin deposition (140). A study by Gao et al. showed that inhibiting autophagy could prevent ferritin deposition (139). Bafilomycin A1 treatment inhibited autophagy while significantly reducing erastin induced ferroptosis (141, 142). GPX4 can also be selectively degraded by chaperone mediated autophagy to promote ferroptosis (143). Although autophagy can prevent cell death by scavenging ROS levels, the role of autophagy in ferroptosis usually involves helping to initiate cell death.

Various studies have also reported the interdependence between autophagy and pyroptosis. In particular, autophagy can reduce endotoxin mediated proinflammatory cell kinase IL-1 β and IL-18 secretion. After Atg16L1 or Atg7 knockout, caspase 1 activity and cytokine secretion were enhanced by LPS (82, 144). A unique feature of autophagy regulating pyroptosis is through the degradation of NLRP3 inflammatory bodies and bacterial pathogens. The NLRP3 inflammasome acts as a sensor of mitochondrial dysfunction, demonstrating the link between mitochondrial damage, autophagy / mitochondrial phagocytosis and inflammation (145). The NLRP3 inflammasome can also be selectively degraded by autophagy, limiting cytokine secretion and reducing pyroptosis (79, 146). On the contrary, NRLP3 can inhibit autophagy (147). Some reports have shown that activation of autophagy can prevent t pyroptosis in a variety of situations (148–151), or inhibition of autophagy can enhance pyroptosis (152, 153). It is worth noting that in order to prevent pyroptosis, autophagosomes often degrade large protein complexes or organelles, rather than just a single signal molecule. Therefore, autophagy produces very different features of pyroptosis regulation compared with ferroptosis and necroptosis.

## Summary

When sepsis associated acute renal injury occurs, all modes of programmed cell death are involved and interrelated. Inflammatory stimulation and increased expression of body death irritants such as TNF-α, the body to produce death receptors (RIPK1 and TRADD, TRAF and cIAPs), and RIPK1 ubiquitinates to form RIPK1 bodies (FADD, caspase-8, cFLIP, RIPK1, and RIPK3) (134, 154–158). The latter cleaves the apoptosis promoter caspase-8 and then activates downstream apoptosis effectors, such as caspase-3, to promote apoptosis (69, 159, 160). Caspase 8 can also degrade cell death stimuli such as TRAIL by autophagy to prevent apoptosis (123). When caspase-8 activity is inhibited or RIPK1 is activated, RIPK1 and RIPK3 are phosphorylated, resulting in necroptosis (123, 161). In addition, during the early stage of inflammation, the body initiates autophagy to protect itself from inflammatory stimulation. Autophagy bodies form to mark the occurrence of autophagy. Autophagy bodies block caspase-8 cleavage by inhibiting RIPK1 bodies, so as to prevent apoptosis. Studies have confirmed that autophagy and necroptosis occur simultaneously, and reducing autophagy flow can enhance necroptosis (129, 138, 162). In addition, autophagy can degrade damaged mitochondria and inhibitors of apoptosis proteins (IAPs) inhibit the occurrence of necroptosis pathways. Autophagy is also involved in the conversion of complexes containing TRIF, RIPK1, RIPK3, and ZBP1, thus promoting necroptosis (131). In addition, autophagy can also interfere with NLRP3 inflammatory bodies, induce the degradation of NLRP3 inflammatory bodies and damaged mitochondria, limit cytokine secretion, inhibit caspase-1 activity, and thereby inhibit the occurrence of pyroptosis (79, 130, 146, 163). In contrast, the NLRP3 inflammasome can also inhibit autophagy to promote pyroptosis (147). Autophagy can also inhibit ferroptosis by inhibiting GPX4 and selectively degrading damaged mitochondria or ferritin. Autophagy can also degrade toxic oxidized lipids that cause damage to cellular DNA and proteins to inhibit ferroptosis (131). When the body continues to be stimulated by infection and inflammation, autophagy decreases, renal tubular cells are damaged, and apoptosis, pyroptosis, necroptosis and inflammatory factors of the circulatory system act together, resulting in acute renal injury.

Sepsis is a multisystem acute infectious disease. The pathological mechanism of acute renal injury is complex and involves many aspects, including renal injury caused by inflammatory factors in the circulatory system. Apoptosis plays a major role in renal injury. However, the study found that apoptosis can not fully explain the serious clinical manifestations of the kidney. With the further study, People have a better understanding of sepsis related acute renal injury. More research confirmed that apoptosis plays a core role in SA-AKI, and non-apoptotic programmed cell death such as pyroptosis necroptosis plays a very important role in SA-AKI. Autophagy dependent cell death also plays an important role in the pathophysiological progression of SA-AKI. Recent studies have reported that ferroptosis also plays an indispensable role in SA-AKI. However, the mechanism of non-apoptotic programmed cell death, especially ferroptosis in SA-AKI is unclear, it needs further research and exploration. Information on the regulatory process between several programmed cell death is limited. This makes the prevention and treatment of SA-AKI more difficult. However, there is limited information on the regulatory process between these types of programmed cell death. This makes the prevention and treatment of SA-AKI more difficult. According to various signaling pathways, the mutual regulation of various types of cell death may occur at different times and developmental stages in the progression of sepsis, as well as in different types of cells. Understanding the interaction between different cell death pathways and clarifying the exact mechanism behind cell death is important to developing potential therapies for the treatment of SA-AKI. This review focuses on the mechanism of programmed cell death in SA-AKI, to provide new insights for the diagnosis and treatment of SA-AKI.

At present, studies have confirmed that there are many kinds of PCD in SA-AKI, but whether these kinds of PCD occur at the same time remains to be further explored. It has been reported that there are three kinds of programmed cell death including apoptosis, pyroptosis and necroptosis in infectious diseases, they have a common upstream regulatory target ZBP1, which is named ZBP1 dependent PANoptosis (164). However, whether this new PCD death model exists in renal disease has not been reported. Whether there is an upstream target that can regulate more PCD modes at the same time needs to be further explored.

## Author Contributions

ZW wrote the paper, collected literature, and produced the chart. JY modified the paper. All authors participated in this review and approved the final version of the manuscript.

## Funding

National Natural Science Foundation of China (No. 81770682), Basic and Frontier Research Program of Chongqing(cstc2017jcyjBX0014), the Chongqing Talent Program Project (cstc2021ycjh-bgzxm0090), Scientific Research Incubation Project of the Third Affiliated Hospital of Chongqing Medical University (KY20078), and Scientific Research Incubation Project of the Third Affiliated Hospital of Chongqing Medical University (KY08031).

## Conflict of Interest

The authors declare that the research was conducted in the absence of any commercial or financial relationships that could be construed as a potential conflict of interest.

## Publisher's Note

All claims expressed in this article are solely those of the authors and do not necessarily represent those of their affiliated organizations, or those of the publisher, the editors and the reviewers. Any product that may be evaluated in this article, or claim that may be made by its manufacturer, is not guaranteed or endorsed by the publisher.

## Abbreviations

SA-AKI: Sepsis-associated acute kidney injury. RTECs
